# Fabrication and thermal properties of tetradecanol/graphene aerogel form-stable composite phase change materials

**DOI:** 10.1038/s41598-018-27038-4

**Published:** 2018-06-11

**Authors:** Boyuan Mu, Min Li

**Affiliations:** 1 Jiangsu Key Laboratory for Construction Materials, Southeast University, Nanjing, 211189 China; 2 International Institute for Urban System Engineering, Southeast University, Nanjing, 210096 China

## Abstract

In this study, tetradecanol/graphene aerogel form-stable composite phase change materials were prepared by physical absorption. Two kinds of graphene aerogels were prepared using vitamin C and ethylenediamine to enhance the thermal conductivity of tetradecanol and prevent its leakage during phase transition. The form-stable composite phase change material exhibited excellent thermal energy storage capacity. The latent heat of the tetradecanol/graphene aerogel composite phase change materials with 5 wt.% graphene aerogel was similar to the theoretical latent heat of pure tetradecanol. The thermal conductivity of the tetradecanol/graphene aerogel composite phase change material improved gradually as the graphene aerogel content increased. The prepared tetradecanol/graphene aerogel composite phase change materials exhibited good thermal reliability and thermal stability, and no chemical reaction occurred between tetradecanol and the graphene aerogel. In addition, the latent heat and thermal conductivity of the tetradecanol/ethylenediamine-graphene aerogel composites were higher than those of tetradecanol/vitamin C-graphene aerogel composites, and the flexible shape of the ethylenediamine-graphene aerogel is suitable for application of the tetradecanol/ethylenediamine-graphene aerogel composite.

## Introduction

Currently, with the limited fossil fuel reserves and the surge in greenhouse gas emissions, satisfying energy demands and reducing pollution will inevitably involve the promotion of green renewable energy technologies. However, most green renewable resources, including solar energy and wind energy, are unreliable due to environmental constraints. Latent heat storage using phase change materials (PCMs) is one of the most viable methods for solving the problems associated with green renewable resources because a large amount of heat energy per unit volume can be stored or released during the phase change process at a nearly constant temperature^1–3^. In general, PCMs can be divided into two categories: inorganic phase change materials and organic phase change materials. Compared to inorganic phase change materials, organic phase change materials have the advantage of higher melting and freezing latent heats, smaller separation, smaller undercooling, lower cost and lower vaporization. In addition, their structures remain unchanged during repeated phase transitions^4^.

Currently, many organic PCMs have been applied in various fields. In solar energy storage and solar cooling, organic PCMs have been used to store solar energy, which is available only during the daytime, for later use^5^. In battery thermal management systems, the operating and environmental temperatures can affect the service life and safety of the batteries, and organic PCMs are required to maintain the operating temperature and lengthen the battery lifetime^6^. In buildings, organic PCMs are incorporated into the building envelope to efficiently reduce electricity and fuel consumption^7^. Moreover, organic PCMs have been investigated for use in the air-conditioning^8^, textile^9^ and food industries^10^. However, two disadvantages have limited the application of organic PCMs. Their low thermal conductivity reduces the rate of heat storage and extraction, restricting practical application. In addition, morphological changes can lead to leakage during the phase change process, which may corrode the contact materials and reduce the thermal cycling stability of the organic PCMs.

PCMs could be incorporated into porous materials to prevent the leakage of PCMs during phase transitions and improve the thermal conductivity. Metal foams^11^, porous carbon-based materials^12^, porous polymers^13^ and porous inorganic materials^14^ are typically employed as porous materials to incorporate PCMs. Porous carbon-based materials are highly attractive as potential PCM supports due to their high thermal conductivity, low density, and chemical stability. In addition, these materials minimize the phase change enthalpy loss resulting from the support materials^15^. Liu *et al*.^16^ reported a dicarboxylic acid eutectic mixture PCM in which expanded graphite was used to suppress the leakage of eutectics. Experiments have shown that the latent heat storage of the eutectics of succinic acid and adipic acid with a molar ratio of 7:3 was 206 J/g, and the thermal property of the final composite PCM remained almost unchanged after 100 thermal cycles. Jiang *et al*.^17^ examined the influence of the mass fraction of expanded graphite (EG) on the thermal management performance of a paraffin/EG composite PCM. Due to the porous structure of EG, the leakage phenomenon of the paraffin during the phase transition decreased as the EG loading increased. In addition, the thermal conductivity of the composite PCM was enhanced with the addition of EG. Polyethylene glycol (PEG) has been introduced into graphene oxide aerogel (GOxA) via vacuum impregnation to prepare a type of porous carbon-based shape-stabilized PCM^18^. GOxA was fabricated using graphene oxide (GO) with a different oxidation degree, and the result demonstrated that a higher degree of oxidation in GO leads to improved shape stability of the composite PCM.

Fatty alcohol is an important organic solid–liquid PCM that has a high thermal energy storage density and low undercooling. In addition, this material is non-toxic and readily available. Tetradecanol (TD) is a type of fatty alcohol, as shown in Table 1. TD possesses a high thermal energy storage density and low undercooling. In addition, TD is non-toxic and readily available. TD exhibits a solid–solid phase transition close to the solid–liquid phase transition. The solid–solid phase transition is also beneficial for thermal energy storage. Graphene aerogel (GA) is a type of three-dimensional (3D) porous derivative of graphene that has the potential for application in many fields, such as energy storage and conversion, catalyst support, water purification, sensors and especially oil adsorption^19^. Due to the hydrophobicity of graphene sheets, GA is typically hydrophobic and oleophilic. Therefore, PCMs can be embedded in the connective pores of the GA via direct physical adsorption^20^. The high thermal conductivity of GA and the large contact surface area between the PCM and graphene sheets can enhance the thermal conductivity of the PCM. In addition, the capillary force of the GA could effectively prevent leakage of the PCM during the phase transition. Therefore, the GA-based composite PCM exhibits high thermal conductivity, high energy storage density, high light-absorbent rate and a form-stable shape that could be used in a battery thermal management system to maintain the temperature of batteries in a normal operating temperature range and could be used in high-power electronics to satisfy the high heat storage and dissipation requirements^21^. Furthermore, a GA-based composite PCM could directly utilize solar irradiation to drive the PCMs for energy conversion and could be used in thermoelectric energy harvesting device for thermal control and storage^22,23^.

**Table 1:** Thermal property of PCMs with similar phase change temperature.

Sample	Melting temperature (°C)	Latent heat of melting (J/g)	Freezing temperature (°C)	Latent heat of freezing (J/g)	Reference
PEG 1000	33.32	143.16	29.67	166.71	^44^
Capric acid	31.04	190.21	27.11	180.34	^45^
Paraffin	38.21	135.46	36.11	158.14	^46^
Tetradecanol	36.2	200.2	33.8	201.5	Current study

However, the extremely difficult methods and harsh reaction conditions that are required for the preparation of PCM/GA composites have limited their application^24,25^. The most popular route for the bulk production of GA is the chemical oxidation-reduction method. The oxidation induces a variety of oxygen-containing functional groups, and the reduction process can lead to the further aggregation or ion doping, which could affect the thermal properties of the GA and TD/GA form-stable composite phase change material (FS-CPCM). In the present study, two types of GA with different content functional groups and microstructures were prepared via strong reductant vitamin C (VC) and weak reductant–ethylenediamine (EDA). TD was selected as the PCM and was impregnated into GA to prepare FS-CPCMs. The influence of the TD adsorption amount on the thermal property of TD/GA FS-PCM was analyzed. The influence of GA, which was prepared by a different reductant, on the thermal storage performance, thermal conductivity, stability and reliability of TD/GA FS-PCMs was investigated and compared.

## Results

### Morphologies and microstructures of GA and TD/GA FS-CPCMs

**Figure 1:**
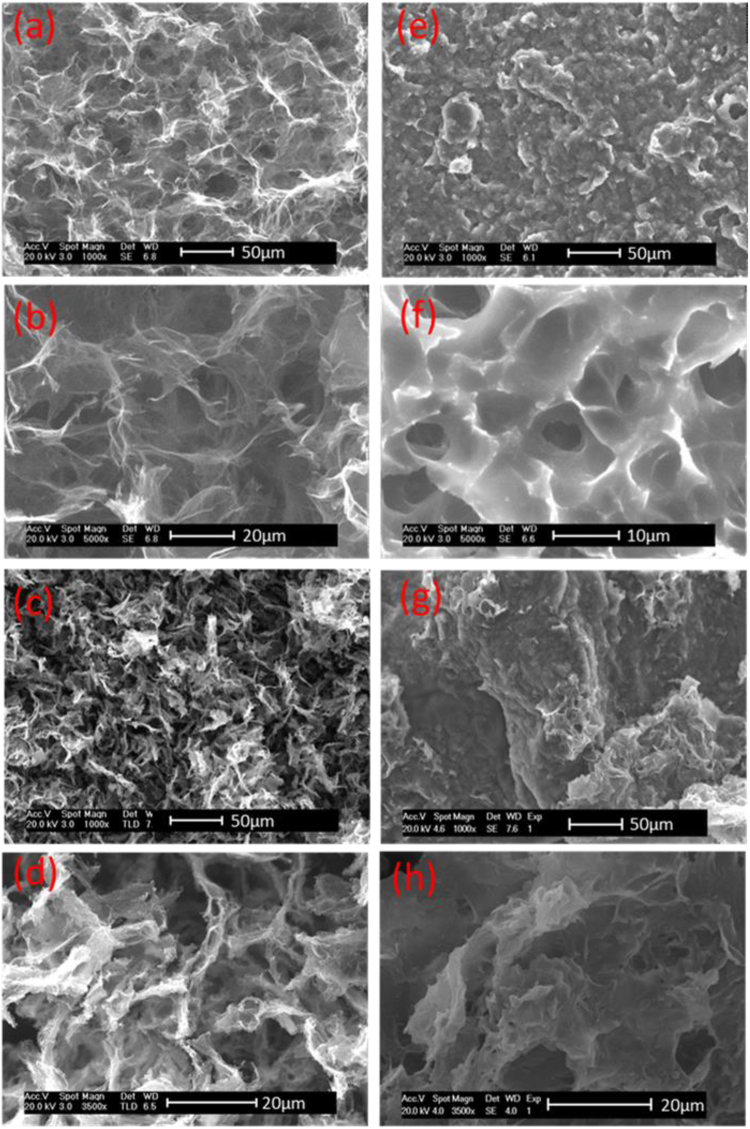
SEM images of EGA (**a** and **b**), VGA (**c** and **d**), TD/EGA-1 (**e** and **f**) and TD/VGA-1 FS-CPCM (**g** and **h**).

The microstructures of GA and TD/GA FS-CPCMs were investigated via SEM analysis and are shown in Fig. 1. Figure 1(a,b) show the morphology of the ethylenediamine-graphene aerogel (EGA), and based on the image, EGA has a 3D porous network structure. The pores were interconnected, and the wall of the EGA consisted of graphene sheets. Moreover, a large number of extremely thin flakes in the EGA could be clearly observed under higher magnification. The size of the interconnected pores in the EGA ranged from several micrometers to over ten micrometers. Organic PCMs can easily absorb into pores of this size in GA^26^. Figure 1(c,d) show the morphology of the vitamin C-graphene aerogel (VGA). The VGA also possessed a 3D porous network structure. However, compared with the EGA microstructures, the thickness of the pore walls consisting of graphene sheets was inhomogeneous and close-packed. The average pore size in the VGA network was less than that in the EGA, which could be attributed to the reduction ability of the reducing agent and GO with different reduction levels can form stable aqueous colloids through electrostatic stabilization in a weak alkaline solution^27,28^. This phenomenon corresponds to the preparation process in the Methods section, which VGA exhibited a greater volume shrinkage than EGA. Figure 1(e–h) show the microstructures of the fracture surfaces of TD/EGA-1 FS-CPCM and TD/VGA-1 FS-CPCM. Most of the pores in Fig. 1(a–d) became narrower or even disappeared. TD uniformly filled the available pore volume in the GA, and TD closely integrated with the graphene sheets. The good wetting property allowed for a more compact combination at the interface of the TD and the graphene sheet. Furthermore, the capillary force could effectively prevent the leakage of TD during phase transition.

**Figure 2:**
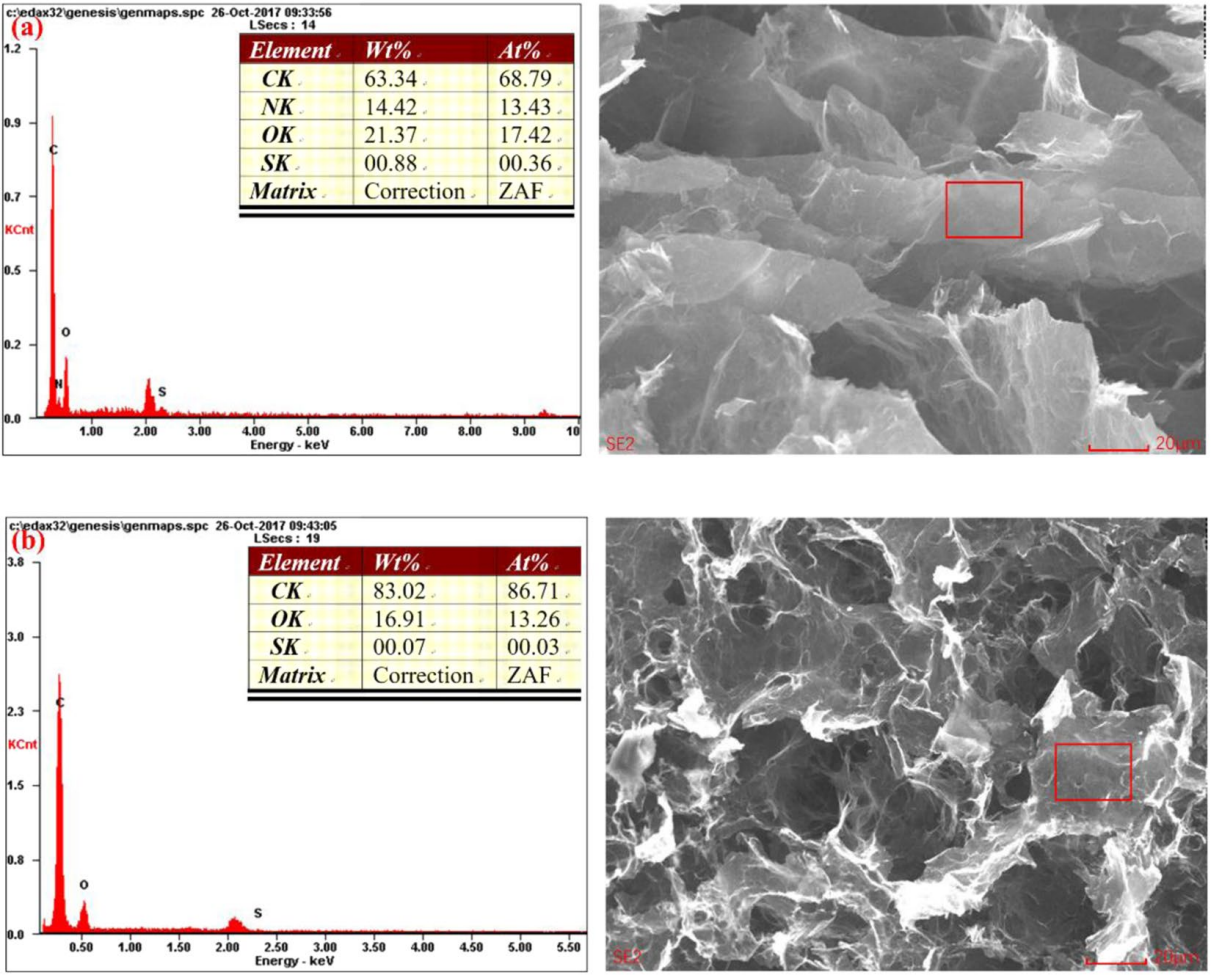
EDS spectra of EGA (**a**) and VGA (**b**).

The elemental characterization of EGA and VGA was determined through EDS analysis and is shown in Fig. 2. The N peaks in Fig. 2(a) indicate the presence of nitrogen-containing functional groups in EGA, and the mass ratio of the oxygen element in EGA was much higher than that in VGA. Therefore, VGA had a higher degree of reduced graphene, and EGA contained more oxygen- and nitrogen-containing functional groups.

**Figure 3:**
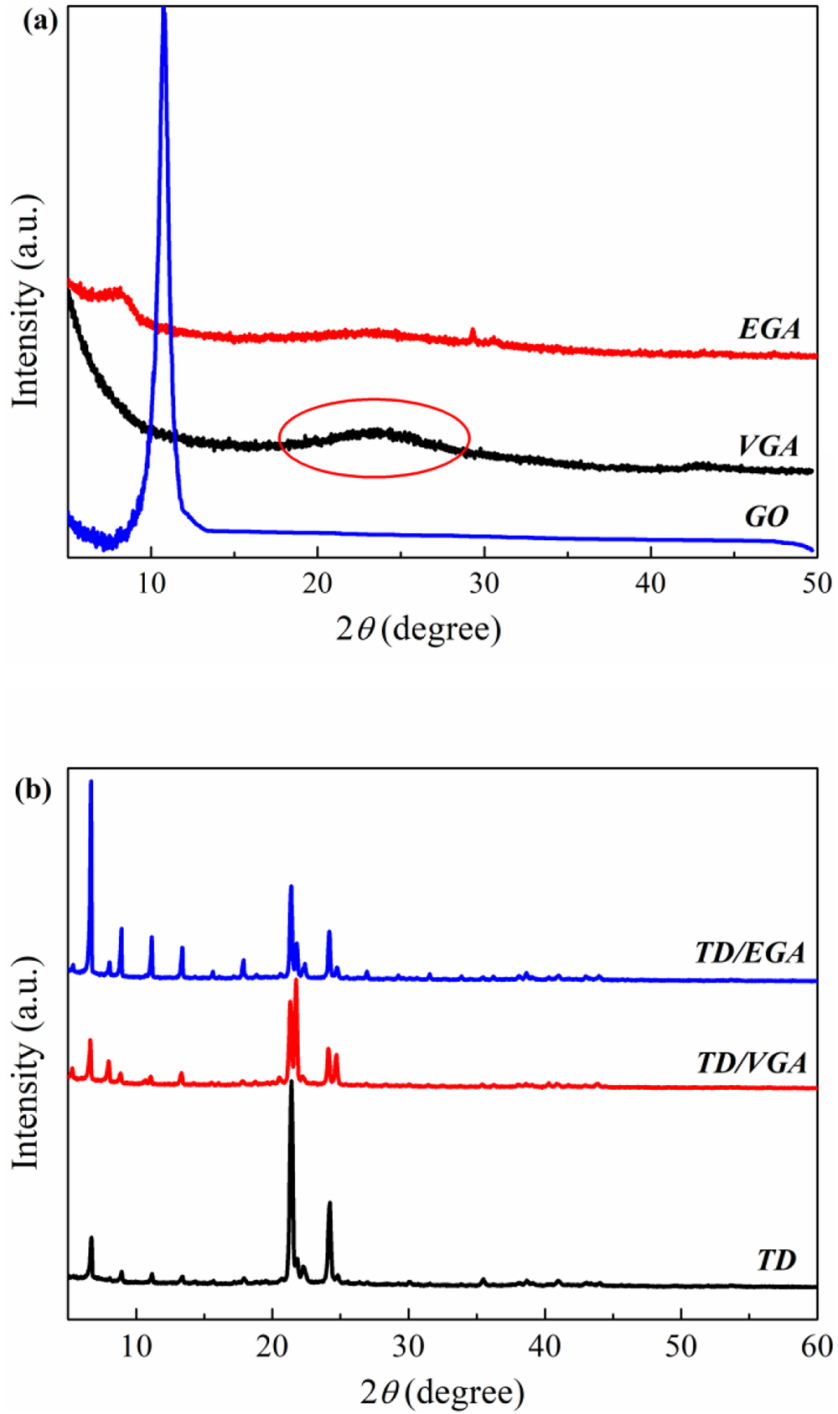
XRD patterns of GA (**a**), TD and TD/GA FS-CPCMs (**b**).

The XRD patterns of GO, EGA and VGA are shown in Fig. 3(a). The XRD pattern of GO, which was oxidized by the flake graphite, contained a prominent peak at 2θ = 11.24°, corresponding to an interlayer spacing of 0.787 nm. This peak indicated that a layered structure was still present in the GO. After the reduction, the peak at 2θ = 11.24° disappeared for EGA and VGA, implying that most of the oxygen functional groups were removed. A new broad peak for VGA was observed at approximately 2θ = 23.6°, corresponding to an interlayer spacing of 0.377 nm. The appearance of the broad peak was caused by the reassembly and stacking of the graphene sheets after the oxygen functional groups were removed. However, no corresponding peak was observed for EGA, indicating that the graphene sheets in the product did not stack due to the steric hindrance from the EDA molecules^29^. These results can explain the differences between the morphologies of VGA and EGA.

The phase structure of the raw TD and TD/GA FS-CPCMs positively influenced the latent heat and phase change temperature. The XRD patterns of pure TD, TD/EGA and TD/VGA FS-CPCM are shown in Fig. 3(b). The three curves are similar. As shown in Fig. 3, the peaks for TD that located at 2θ = 21.2°, 21.7°, and 24.1° were strong diffraction peaks, and the peak at 2θ = 24.7° was weak. In the curves for TD/EGA and TD/VGA FS-CPCM, the same diffraction peaks were also observed (i.e., 2θ = 21.7°, 24.1°, 21.2° and 24.7°). However, these peaks were weaker than those for pure TD, which was most likely due to the defective crystalline state of TD in the GA pores^30^. In addition, closer observation revealed that the peaks at 2θ = 21.7° and 24.7° for TD/VGA FS-CPCM became stronger than those for TD and TD/EGA FS-CPCM. Solid fatty alcohol has two phases (i.e., metastable hexagonal orthorhombic solid phase (S_HEX_) and orthorhombic solid phase (S_ORT_)). The TD in the TD/GA FS-CPCMs was strongly absorbed by GA and constrained in the GA pores. Based on SEM analysis, VGA possessed a smaller pore size than EGA, and the pores hindered the transition between S_HEX_ and S_ORT_. The pure TD and TD/EGA FS-CPCM preferred the S_ORT_, and the TD in the TD/VGA FS-CPCM primarily existed as S_HEX_^31^. Therefore, the XRD curves of TD/EGA and TD/VGA FS-CPCM exhibited the previously mentioned differences.

### Chemical characterization of GA and TD/GA FS-CPCMs

**Figure 4:**
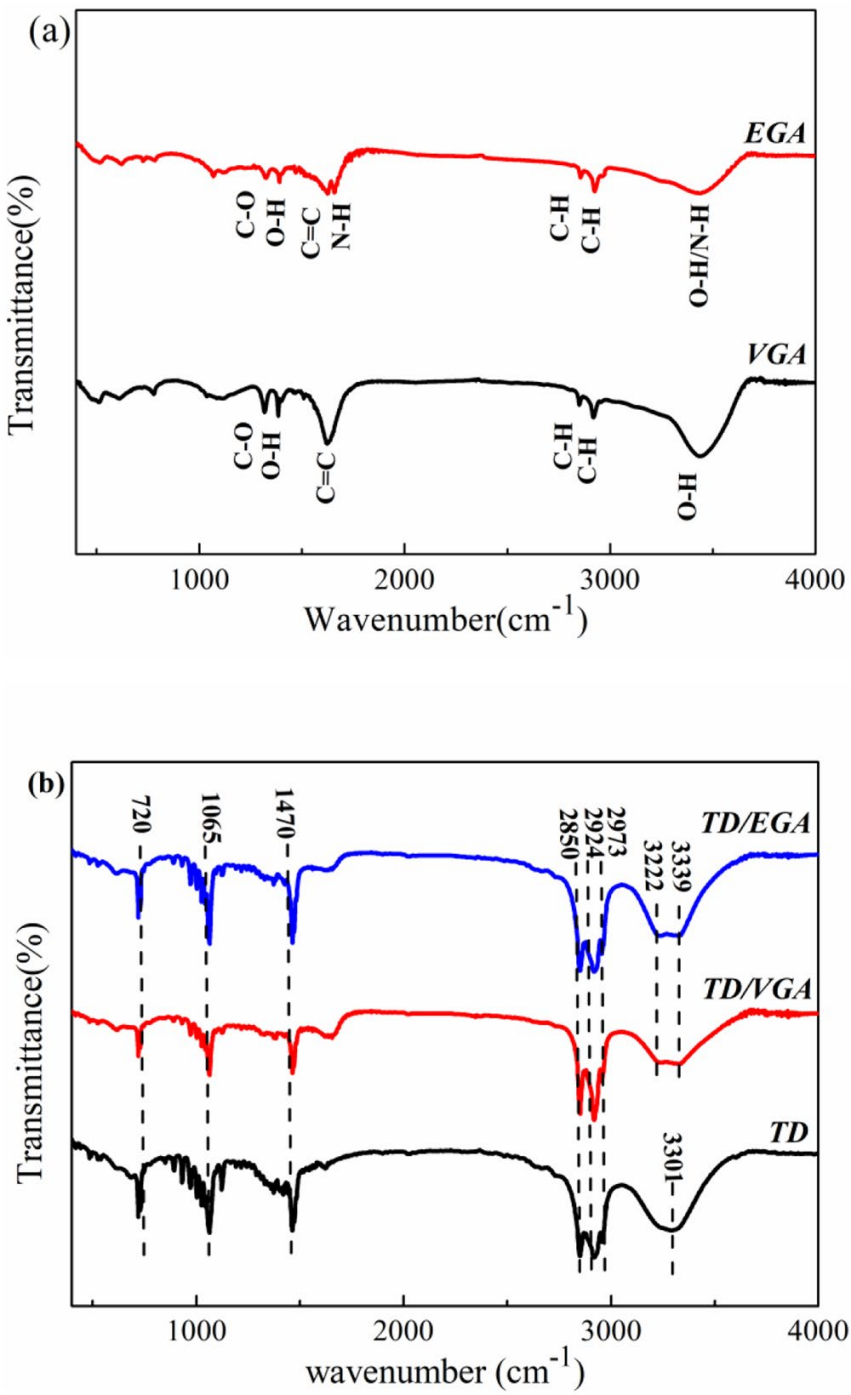
FTIR spectra of GA (**a**) and TD/GA FS-CPCMs (**b**).

The FTIR spectrum was recorded to characterize the structure of GA, TD and TD/GA FS-CPCMs. As shown in Fig. 4(a), in the FTIR spectrum of VGA, the peak at 3431 cm^−1^ corresponded to the stretching vibration of O-H group. The stretching vibration peak of C=C was observed at 1626 cm^−1^. The peaks at 1313 cm^−1^ and 1385 cm^−1^ corresponded to C-O and O-H bending vibrations^19^. The peaks at 2858 cm^−1^ and 2928 cm^−1^ indicated that the C-H bond was located at the edge of the graphene sheet. For EGA and VGA, their FTIR spectra were very similar. The range of the peak at 3474 cm^−1^ for EGA broadened due to overlapping with the peak associated with the O-H and N-H stretching vibrations. The weak peaks at 1633 cm^−1^ and 1647 cm^−1^ corresponded to the deformation vibration peak of N-H and the stretching vibration peak of C=C^19,32^.

The FTIR spectra of TD, TD/EGA and TD/VGA FS-CPCM are shown in Fig. 4(b). The broad band centered at 3301 cm^−1^ in the spectrum of TD was caused by the stretching vibration of the O-H bond in alcohols. The peaks at 2973 cm^−1^, 2924 cm^−1^ and 2850 cm^−1^ corresponded to the stretching vibrations of −CH due to −CH_3_ and CH_2_, respectively. The C-H bending vibration peak was observed at 1470 cm^−1^ and 720 cm^−1^. The stretching vibration peak of located at 1065 cm^−1^ was the C–O group^33^. The absorption peaks in the FTIR spectra of TD/GA FS-CPCMs and TD were very similar. Only one difference between TD/GA FS-CPCMs and TD was observed. The two peaks at 3222 cm^−1^ and 3339 cm^−1^ for FS-CPCMs were due to the stretching vibration of the O-H group of GA or the hydrogen bond formed by the hydrogen of the carbon chain of TD and GA. The results in Fig. 4(b) indicated that the TD was only physically absorbed into VGA and EGA. In addition, no chemical interaction existed between the TD and GA.

### Thermal conductivity characteristics of GA and the TD/GA FS-CPCMs

**Figure 5:**
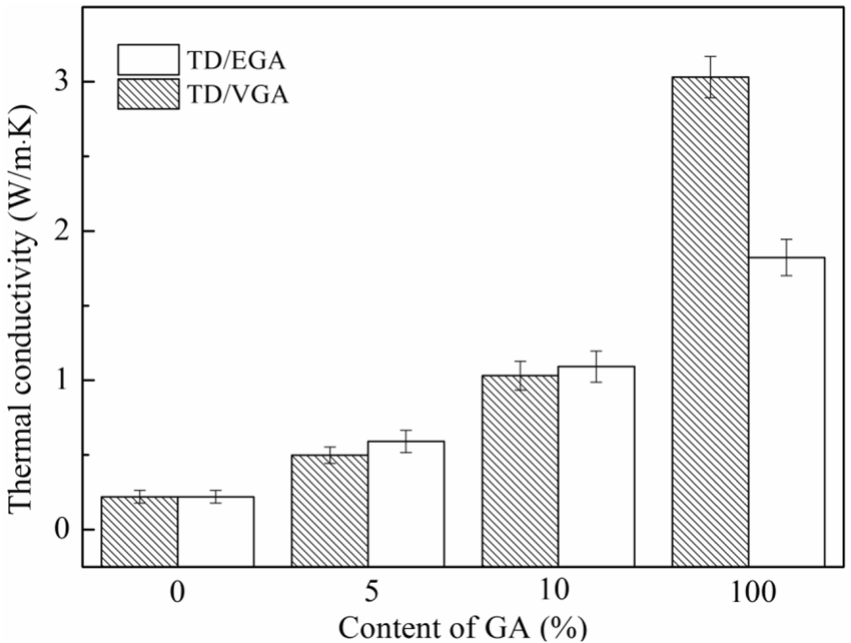
Thermal conductivity of the TD, TD/GA FS-CPCM and GA.

The thermal conductivity of TD, TD/GA FS-CPCMs and GA is shown in Fig. 5, and the thermal conductivity of VGA and EGA was 3.031 W/m·K and 1.823 W/m·K, respectively. Based on the results, the thermal conductivity of the TD/GA FS-CPCMs increased gradually as the GA content increased. The thermal conductivity of pure TD was 0.221 W/m·K. The thermal conductivities of the TD/EGA FS-CPCMs containing 5 wt.% and 10 wt.% GA were 0.591 W/m·K and 1.092 W/m·K, respectively. These values were 167% and 394% higher than that of pure TD, respectively. However, the thermal conductivities of the TD/VGA FS-CPCMs were 0.498 W/m·K and 1.031 W/m·K (i.e., 125% and 367% higher than that of pure TD, respectively). These results indicated that the thermal conductivity of the TD/GA FS-CPCMs was substantially improved by the GA.

As shown in Fig. 5, although the thermal conductivity of VGA was much higher than that of EGA, the thermal conductivity of TD/EGA FS-CPCM was higher than that of TD/VGA FS-CPCM. Although the reducibility of EDA was weak, EGA contained more functional groups (−OH, −C=O, −NH). The functionalized GA altered the hybrid and vibrational modes^34^, and the vibration coupling degree between the carbon atoms of EGA and TD increased. Therefore, the interfacial thermal scattering was effectively reduced^35,36^. The heat flux at the interface of the transmission process produced a smaller energy loss. Therefore, the interfacial thermal conductance increased due to the functional groups, and the thermal conductivity of TD/EGA FS-CPCM was higher than that of TD/VGA FS-CPCM.

### Thermal properties of GA and the TD/GA FS-CPCMs

**Figure 6:**
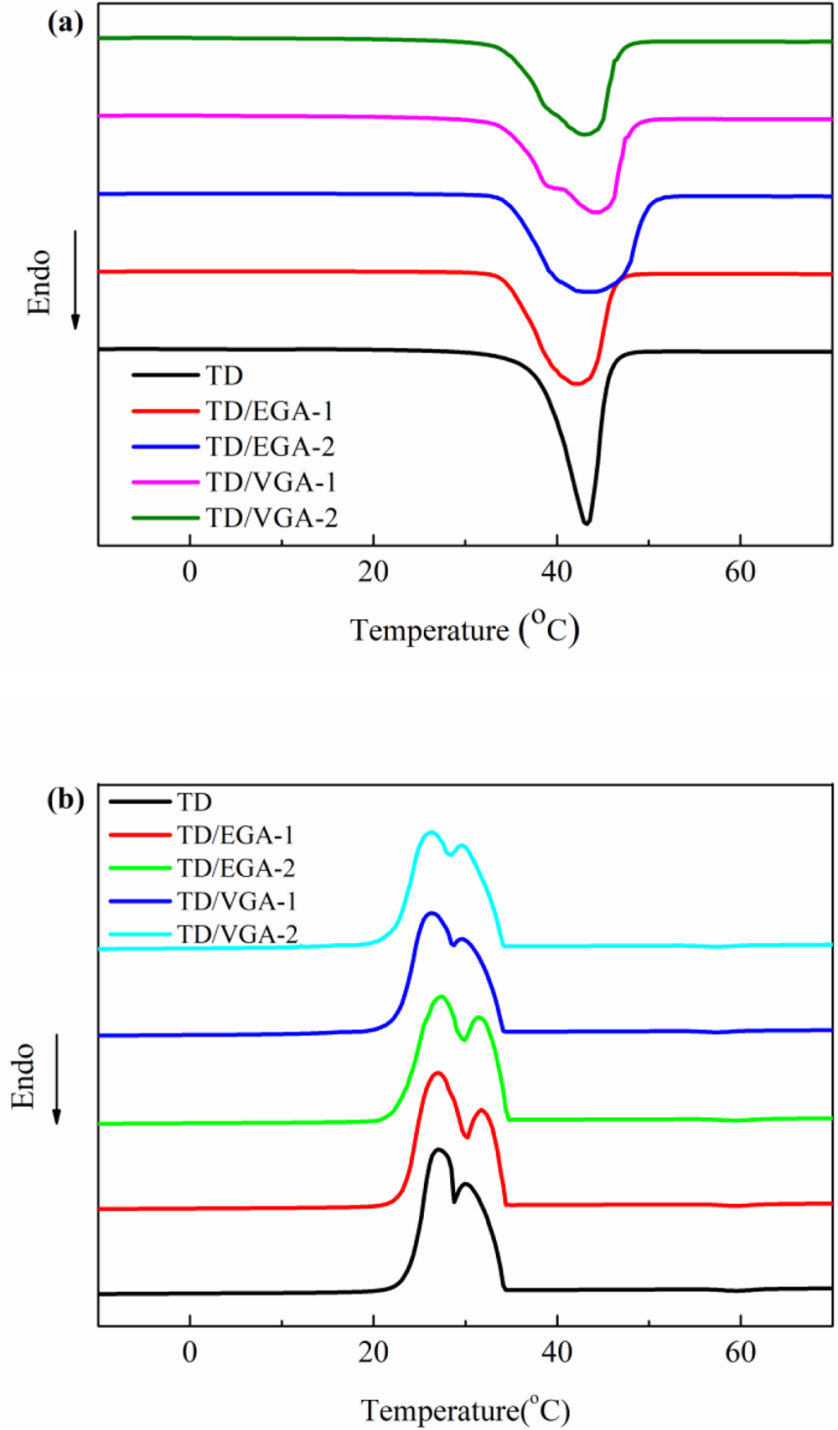
DSC curves of TD, TD/GA FS-CPCMs: (**a**) Melting and (**b**) Freezing processes.

The thermal energy storage properties of TD, TD/EGA and TD/VGA FS-CPCMs were investigated using DSC. The results are shown in Fig. 6(a,b). Pure TD exhibited one endothermic peak and two exothermic peaks. As previously mentioned, solid fatty alcohol has two phases that include metastable S_HEX_ and S_ORT_. The higher peak was the exothermic peak due to the transformation from the liquid form to S_HEX_, and the lower peak was the exothermic peak due to the transformation from S_HEX_ to S_ORT_^33^. Nevertheless, the two-phase transition process occurring very closely resulted in one endothermic peak^37^. The results in Fig. 6(a,b) indicate that the DSC curves of both TD/EGA and TD/VGA FS-CPCMs were similar to that of TD. Furthermore, it was obvious that, compared with that of pure TD, the endothermic peak of the TD/GA FS-CPCMs was wider and less intense, and the area of the endothermic and exothermic peaks decreased as the GA content increased. The phase change peak of pure PCM is higher and narrower than the phase change peak of a mixed PCM^38^.

Based on DSC analyses, the melting temperature (*T*_*m*_), freezing temperature (*T*_*f*_), melting latent heat (Δ*H*_*m*_), freezing latent heat (Δ*H*_*f*_), and theoretical latent heat (Δ*H*_*m*_^*T*^; Δ*H*_*f*_^*T*^) of pure TD and TD/GA FS-CPCMs were determined, and these results are summarized in Table 2. *T*_*m*_ and *T*_*f*_ of pure TD were 36.2 °C and 33.8 °C, respectively. The *T*_*m*_ and *T*_*f*_ of TD/GA FS-CPCMs were similar to those of pure TD because TD plays an important role in the phase transition process. In addition, no chemical interaction occurred between TD and GA. Furthermore, compared with pure TD, a decrease of T_m_ and slight increase in T_f_ due to the increasing GA content are shown in Table 2. This phenomenon indicated that the undercooling decreased as GA was added. The pores in GA acted as a heterogeneous nucleation center that promoted crystallization of the TD dispersed in the GA.

**Table 2:** Thermal properties of TD and TD/GA FS-CPCMs.

Sample	Melting process			Freezing process		
	T_m_ (°C)	ΔH_m_ (J/g)	ΔH_m_^T^ (J/g)	T_f_ (°C)	ΔH_f_ (J/g)	ΔH_f_^T^ (J/g)
TD	36.2	200.2	/	33.8	201.5	/
TD/EGA-1	35.4	191.8	190.2	34.0	193.3	191.4
TD/EGA-2	35.4	177.7	180.2	34.1	180.2	181.4
TD/VGA-1	35.1	190.1	190.2	33.8	192.6	191.4
TD/VGA-2	34.9	177.6	180.2	33.9	178.7	181.4

The Δ*H*_*m*_ and Δ*H*_*f*_ of pure TD were 200.2 J/g and 201.5 J/g. When the EGA was mixed with the TD at 5 wt.% and 10 wt.%, the Δ*H*_*m*_ and Δ*H*_*f*_ were 191.8 J/g and 193.3 J/g and 177.7 J/g and 180.2 J/g, respectively. Using the same mass fractions, the Δ*H*_*m*_ and Δ*H*_*f*_ of TD/VGA were 190.1 J/g and 192.6 J/g and 177.6 J/g and 178.7 J/g, respectively. The loss of the latent heat was due to some mass fraction of TD being replaced by the GA. Using simple mixing theory, the theoretical data are defined as follows:

1 $${\rm{\Delta }}{H}^{T}={\rm{\Delta }}{H}_{TD}\ast (1-X)$$

where Δ*H*^*T*^ is the theoretical latent heat of TD/GA FS-CPCMs, Δ*H*_*TD*_ is the latent heat of pure TD, and *X* is the mass fraction of GA. As shown in Table 2, the experimental data were similar to the theoretical latent heat when the GA content was 5 wt.%. However, the experimental data for TD/GA FS-CPCMs with 10 wt.% GA were less than the theoretical latent heat. The variation in the latent heat due to the GA mass fraction was due to the thermal conductivity and phase structure of FS-CPCM. As the mass fraction of GA increased, the increase in the thermal conductivity of FS-CPCM thermally accelerated the evaporation of pure TD during the kneading process^39,40^, and the defective crystalline state of TD in GA had an adverse effect on the latent heat of TD. As shown in Table 2, TD/VGA FS-CPCM exhibited a lower undercooling and phase change latent heat than TD/EGA FS-CPCM with the same GA content because VGA exerted a larger effect on the crystallization of TD than EGA.

### Thermal reliability characteristics of the TD/GA FS-CPCMs

**Figure 7:**
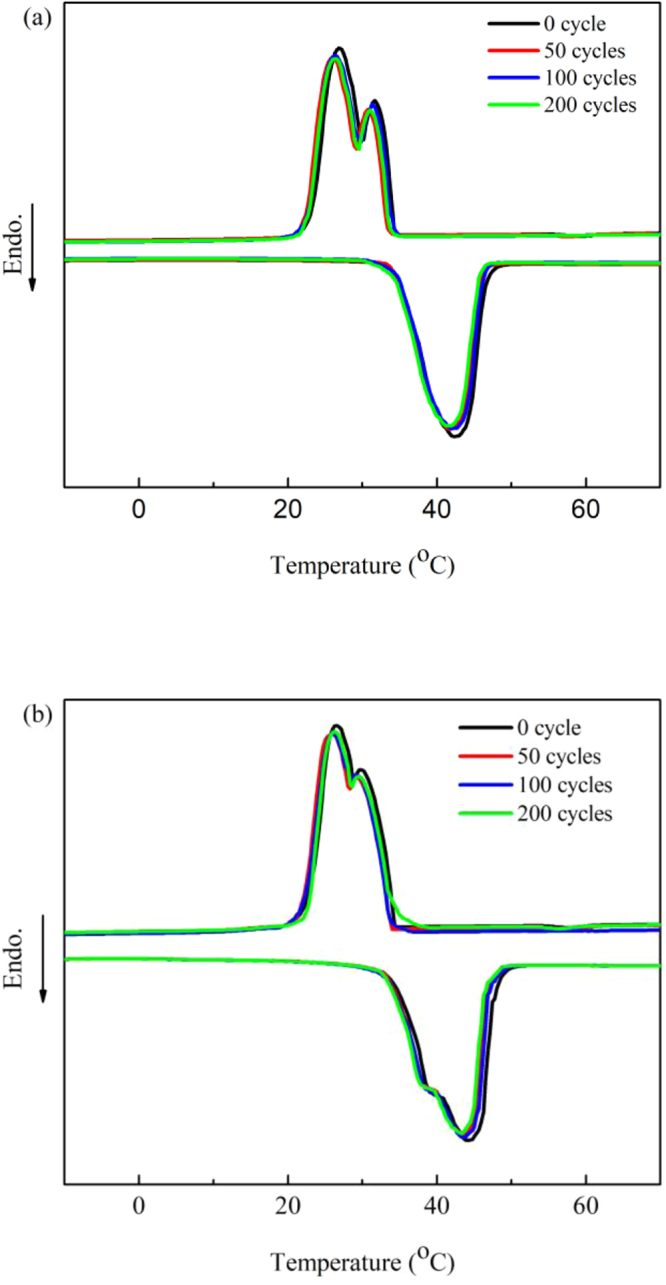
DSC curves for TD/GA FS-CPCM before and after thermal cycling: (**a**) TD/EGA-1 and (**b**) TD/VGA-1.

The thermal reliability of the TD/EGA-1 and TD/VGA-1 FS-CPCMs after various accelerated thermal cycles is shown in Fig. 7, and the thermal properties of the TD/GA FS-CPCMs are summarized in Table 3. As shown in Fig. 7, after 50, 100, and 200 thermal cycles, the DSC curves were similar to that of the TD/GA FS-CPCM prior to the thermal cycles, and the phase change temperature (*T*_*m*_ and *T*_*f*_) of both TD/EGA-1 and TD/VGA FS-CPCMs after the thermal cycles was slightly higher than that without thermal cycling. The largest decrease in Δ*H*_*m*_ for /EGA-1 and TD/VGA-1 FS-CPCMs after thermal cycles was 6.8% and 4.6%, respectively, and the largest decrease in Δ*H*_*f*_ was 6.8% and 4.7%, respectively. These results indicate that the changes in the phase change temperature and latent heat value were acceptable for practical application. Therefore, TD/GA FS-CPCM exhibited good thermal reliability after 200 thermal cycles.

**Table 3:** Thermal properties of the TD/GA FS-CPCMs before and after thermal cycling.

Sample	Melting process		Freezing process	
	T_m_ (°C)	ΔH_m_ (J/g)	T_f_ (°C)	ΔH_f_ (J/g)
TD/EGA-1	35.4	191.8	34.0	193.3
TD/EGA-1 50cycle	34.8	178.8	33.2	180.7
TD/EGA-1 100cycle	34.9	179.3	33.7	182.5
TD/EGA-1 200cycle	34.5	178.9	33.3	180.1
TD/VGA-1	35.1	190.1	33.8	192.6
TD/VGA-1 50cycle	34.8	181.3	33.5	183.6
TD/VGA-1 100cycle	34.7	183.5	33.6	183.7
TD/VGA-1 200cycle	34.6	181.6	33.7	184.2

### Thermal stability characteristics of the TD/GA FS-CPCMs

**Figure 8:**
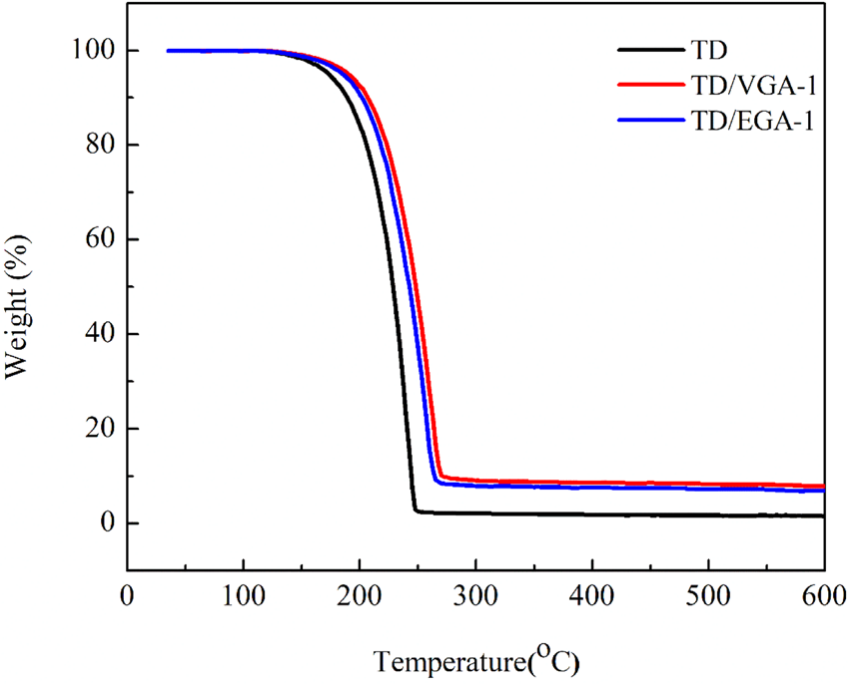
TG curves of TD and TD/GA FS-PCMs.

Thermal stability is a significant parameter for evaluating the properties of TD/GA FS-PCMs that are used for heat energy storage. The thermal stability of the TD/EG FS-CPCMs was investigated by TG, and the curves are shown in Fig. 8. The weight loss for TD began at 137 °C, and almost no residual remains at 247 °C. Therefore, TD experienced a simple decomposition reaction. For the TD/GA FS-CPCMs, the weight loss trend was similar. The decomposition began at ~147 °C and ends at ~272 °C. The residue of the TD/GA FS-PCMs was in good agreement with the GA loading in the TD/GA FS-PCMs. The thermal stability of TD/GA FS-CPCM was higher than that of TD. In addition, the lower mass fraction of the TD/EGA FS-PCM residues corresponded to the decomposition of nitrogen- and oxygen-containing functional groups in EGA.

### Heat storage and release property of GA and TD/GA FS-CPCMs

**Figure 9:**
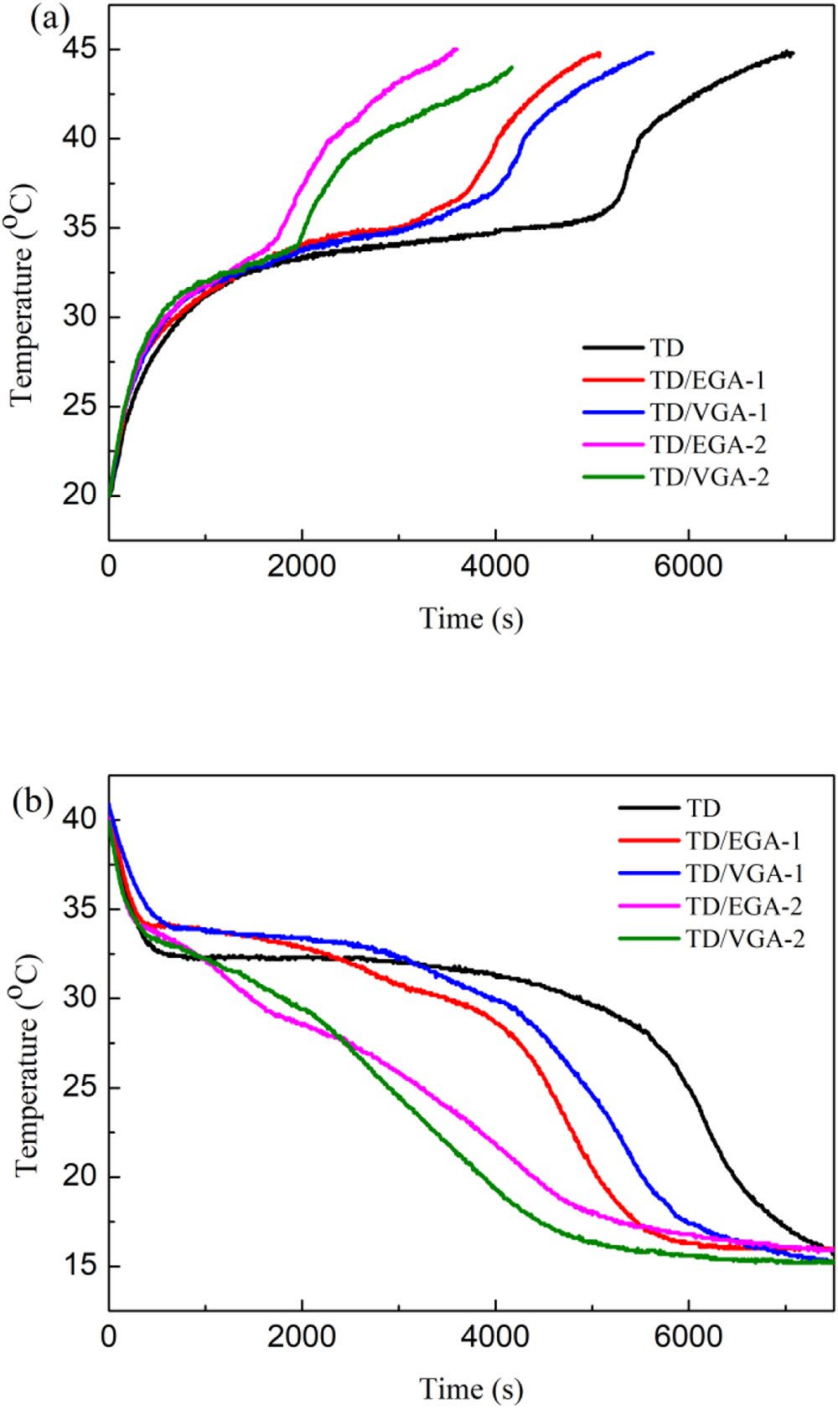
Heating (**a**) and cooling (**b**) curves of TD and TD/GA FS-CPCMs.

The temperature–time curves are shown in Fig. 9 to characterize the heat storage and release properties of TD and TD/GA FS-CPCMs. As shown in Fig. 9(a), 7000 s were required to heat pure TD from 25 °C to 45 °C, and a temperature plateau was observed at 34–37 °C, indicating that TD experienced a phase transition at 34–37 °C. When the mass fraction of GA increased from 5 wt.% to 10 wt.%, the temperature plateau was short, and the heating rates from 25 °C to 45 °C increased. In addition, TD/EGA FS-CPCM had a slightly higher rate of increase than TD/VGA FS-CPCM. Based on the results in Fig. 9(b), when the temperature decreased from 40 °C to 15 °C, 7500 s was required for TD, and the temperature plateau appeared at 30–34 °C, which is the phase change temperature of TD. Both TD/GA-1 FS-CPCMs and TD/GA-2 FS-CPCMs exhibited no obvious phase change temperature plateau. As the mass fraction of GA increased from 5 wt.% to 10 wt.%, the cooling time from 40 °C to 15 °C decreased. The curves for TD/EGA and TD/VGA FS-CPCM were similar when the same amount of GA was added. The shorter melting and cooling times of TD/GA FS-CPCMs were caused by two factors. The thermal conductivities of TD/GA FS-CPCMs were larger than that of TD, and the latent heat of TD/GA FS-CPCMs was less than that of TD^41,42^. Based on previous DSC analysis, the effect of a low mass fraction of GA on the latent heat was limited. Therefore, the higher thermal conductivity was the primary cause of the shorter heating and cooling times of TD/GA FS-CPCMs than those of TD.

## Discussion

In this study, two types of TD/GA FS-CPCMs with the GA mass fractions of 5 wt.% and 10 wt.% were prepared. The properties of the TD/VGA FS-CPCMs and TD/EGA FS-CPCMs were studied and compared in detail. The conclusions are as follows:

1. EGA and VGA exhibited 3D porous network structures. The TD was distributed uniformly in the pores of the GA and closely integrated into the graphene sheet. Compared with EGA, the graphene sheet of the VGA was close-packed, and the pores of VGA were smaller than those of EGA. This structure changed the crystal form and hindered the solid–solid phase transition of TD in the TD/VGA FS-CPCM. The chemical structures of TD did not change during the physical adsorption process that was employed to prepare the TD/GA FS-CPCMs.
2. The thermal conductivity of TD was substantially improved by GA and increased as the GA mass fraction increased, which was confirmed by comparing the temperature–time curves of TD/GA FS-CPCM with those of pure TD. The thermal conductivity of TD/EGA FS-CPCM was higher than that of TD/VGA FS-CPCM, and this difference decreased as the GA content increased.
3. The TD/GA FS-CPCMs exhibited excellent thermal properties, and the Δ*H*_*m*_ and Δ*H*_*f*_ of TD/EGA and TD/VGA FS-CPCMs with 5 wt.% GA were similar to the theoretical latent heat of pure TD. Furthermore, the results of the thermal cycles and thermal gravimetric analysis indicate that the prepared TD/GA FS-CPCMs possessed good thermal reliability and thermal stability.

Therefore, the TD/EGA FS-CPCMs exhibited better thermal properties than the TD/VGA FS-CPCMs, and the variable shape of EGA was suitable for the application of TD/GA.

## Methods

### Materials

Graphite powder was purchased from Qingdao Jinrilai Graphite Co., China. Potassium permanganate (KMnO_4_, AR grade), sulfuric acid (H_2_SO_4_, 98%) and 1-tetradecanol (TD, CP grade) were obtained from Sinopharm Chemical Reagent Co., Ltd., China. Hydrogen peroxide (H_2_O_2_, 29–32% w/w AR grade), sodium nitrate (NaNO_3_, AR grade) and vitamin C (VC, AR grade) were purchased from Chengdu Kelong Chemical Reagent Factory, China. Ethylenediamine (EDA) was purchased from Shandong West Asia Chemical Industry Co., Ltd., China.

### Preparation of GA and TD/GA FS-CPCMs

In this study, a modified Hummers’ method was employed to prepare GO^43^. Briefly, graphite powder, H_2_SO_4_ and NaNO_3_ were mixed and precooled for 30 min in a flask, and then, KMnO_4_ was slowly added to the flask. After stirring for 2 h below 5 °C and stirring for 0.5 h at 35 °C, the flask was moved to a water bath and stirred at 95 °C for 15 min followed by the slow addition of deionized water. The reaction was completed after a certain amount of deionized water was added dropwise, and 30% H_2_O_2_ was added to the flask to cool and remove the residual KMnO_4_. Finally, the solution was centrifuged, washed, and dried to form GO.

**Figure 10:**
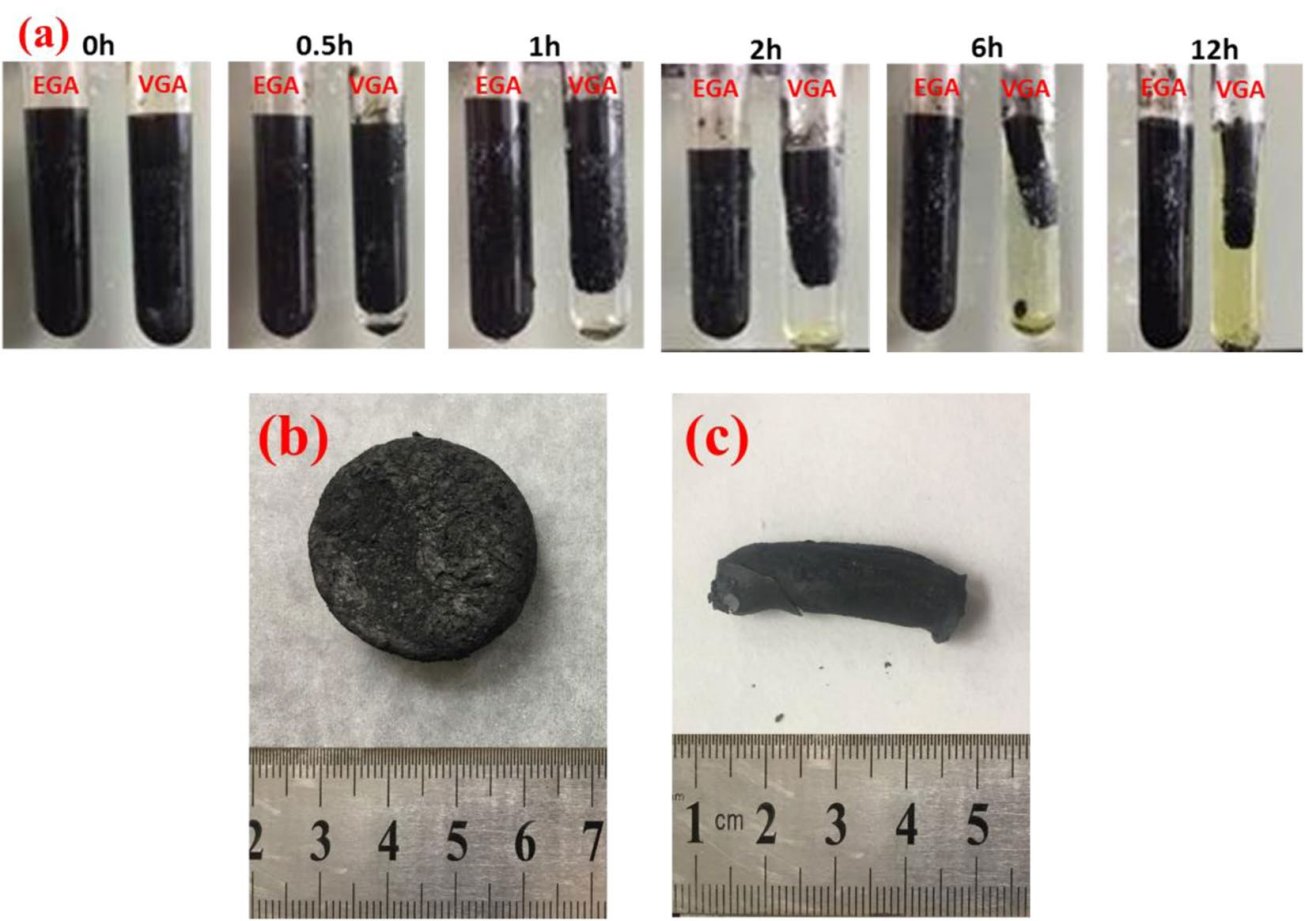
Preparation process for the GA (**a**) as well as photographs of EGA (**b**) and VGA (**c**).

The synthesis process for the graphene hydrogel is shown in Fig. 10(a). A 5 mg/mL homogeneous graphene oxide aqueous solution was prepared by dissolving 0.1 g of GO in 20 mL of water followed by ultrasonic treatment for 0.5 h. Then, 0.2 g of VC and 200 μl of EDA were added to the graphene oxide aqueous solution in two separate test tubes, which corresponded to a VC:EDA molar ratio of 1:2.6. VC-graphene and EDA-graphene hydrogels were prepared after allowing the mixed solution to stand at 70 °C for 12 h. The two types of graphene hydrogel were dialyzed several times using deionized water to remove residual reductant molecules and impurities, and the graphene hydrogel was freeze-dried for two days to prepare the GA. Finally, the two types of GA were heat treated at 60 °C in an argon atmosphere for 24 h. For convenience, the GA that was reduced by VC and EDA are referred to as VGA and EGA, respectively. As shown in Fig. 10(b), the VGA exhibited a remarkable volume shrinkage, and the shape of the VGA was cylindrical. In contrast, EGA exhibited negligible volume shrinkage, and the shape of the EGA was determined by the container. Therefore, EGA has a wider application range than VGA.

TD was coated on the surface of the GA and heated to 60 °C in a vacuum oven for 1 h (i.e., GA mass content of 3 wt.%, 5 wt.%, and 10 wt.%,).

**Figure 11:**
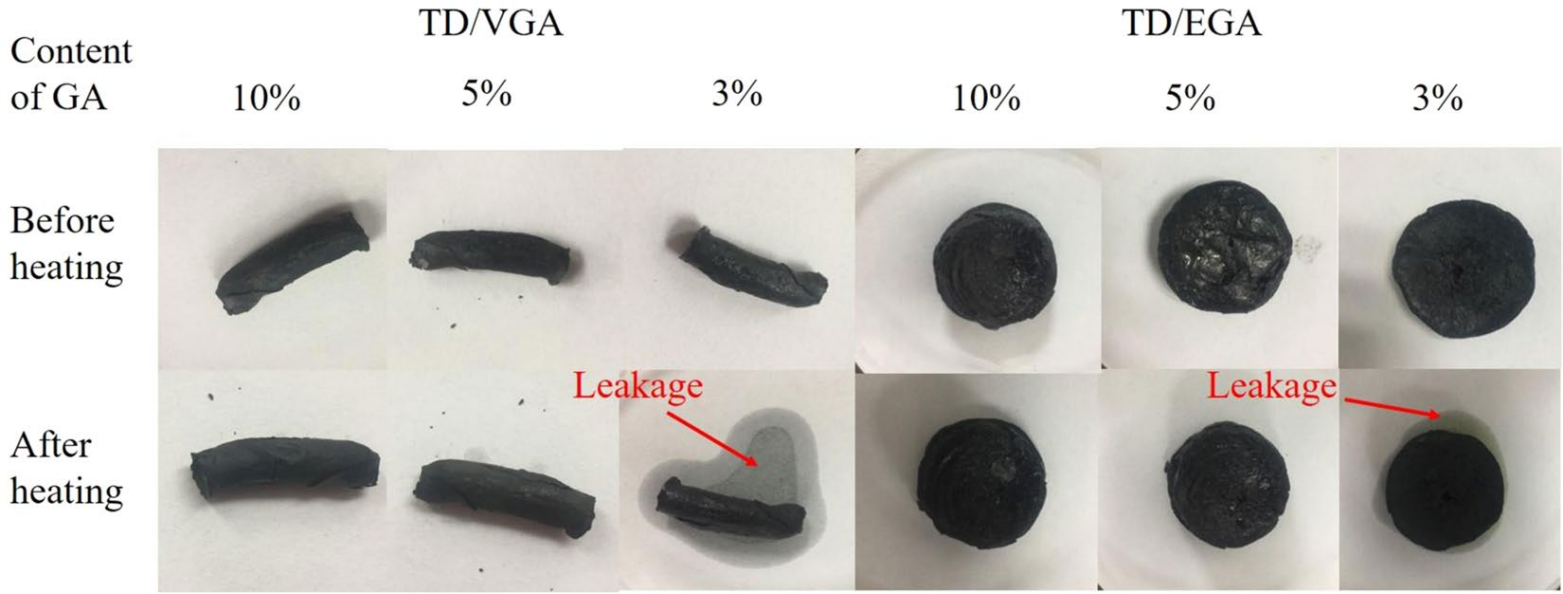
Images of TD/GA FS-CPCMs before and after the leakage tests.

To determine the optimal mass ratio of the TD/GA FS-CPCM, seepage tests were performed. The FS-CPCMs were placed in filter papers and heated in an oven at 60 °C for 1 h to thoroughly melt the composite. Then, the filter papers were observed, as shown in Fig. 11. The lowest loading of GA in the FS-CPCMs was 5 wt.%. The sample formulations are designated in Table 4.

**Table 4:** Formulations for the preparation of TD/GA FS-PCMs.

Sample	GA (g)	TD (g)	Content of GA (%)
TD/EGA-1	1.2374	23.5106	5
TD/EGA-2	1.5603	14.0427	10
TD/VGA-1	1.4251	27.0769	5
TD/VGA-2	1.6783	15.1047	10

### Characterizations

The surface morphology of GA as well as the impregnation and combination states of the TD/GA FS-CPCMs were obtained using a Quanta FEG250 scanning electron microscope (SEM, Sirion 200, FEI Company, Netherlands) at 5 kV. Prior to the examination, all samples were treated by gold spraying. The elemental analysis was accomplished via an energy dispersive spectroscope (EDS) attached to an SEM. Fourier transform infrared spectroscopy (FTIR) was used to characterize the functional groups of the pure TD and TD/GA FS-CPCMs, and the FTIR spectrometer (Magna 750, Nicole Company, USA) scanned from 4000 to 400 cm^−1^ using a KBr pellet. The X-ray diffraction (XRD) patterns of the GA and TD/GA were obtained using an X-ray diffractometer (Smart Lab 3, RIGAKU, Japan) over a 2θ range of 5–70°. A differential scanning calorimeter (DSC, DSC 200 F3 Maia, NETZSCH, Germany) was used to analyze the thermal properties of pure TD and TD/GA FS-CPCMs under a nitrogen atmosphere with a nitrogen flow rate of 60 ml min^−1^. The samples were heated from −20 °C to 70 °C at a ramp rate of 5 °C/min. The analyzer was calibrated using indium (99.9%) prior to the experiment. The temperature fluctuation was ±0.1 °C, and the accuracy of the DSC device was 0.1 µW. All samples were tested three times, and the averages and maximum deviations of the phase change temperature and latent heat were calculated to be ±0.2 °C and ±0.5 J/g, respectively. The thermal reliability of the TD/GA composite was studied by placing the sample in a sealed glass bottle, which was heated in an oven at 80 °C for 20 min followed by cooling at room temperature for 20 min prior to use in the 50, 100 and 200 heating–cooling cycle tests. A thermal gravimetric analyzer (Netzsch STA449 F3, Germany) with a heating rate of 10 °C/min from room temperature to 600 °C under an argon atmosphere was used to study the thermal stability. The thermal conductivities of the GA, TD and TD/GA FS-CPCMs at 20 °C were determined using a thermal constants analyzer (TPS2500, Hot Disk AB Company, Sweden). The transient plane source method was adopted as the measurement method. The specimen was a cylinder with a height of 20 mm and a diameter of 30 mm, and the average value of the three data points was taken as the final value of the thermal conductivity. The standard deviation was calculated and indicated in the figure. The precision of the hot disk is ±3%. The heat storage and release properties of the TD and TD/GA FS-CPCMs with different GA mass fractions were studied using a multi-channel temperature recorder (TOPRIE-TP700, Bost, China). In this test, 10 g of the sample were placed in a test tube, which was placed in a water bath at 45 °C. The thermocouple of the temperature recorder was inserted into the center of the specimen, and the temperature was recorded once every 5 s. The samples were placed in a water bath at a temperature of 15 °C to begin the cooling process after completion of the heating process.
